# Systematic Analysis of the Molecular Mechanism Underlying Decidualization Using a Text Mining Approach

**DOI:** 10.1371/journal.pone.0134585

**Published:** 2015-07-29

**Authors:** Ji-Long Liu, Tong-Song Wang

**Affiliations:** 1 College of Veterinary Medicine, South China Agricultural University, Guangzhou 510642, China; 2 Department of Biology, Shantou University, Shantou 515063, China; University of Science and Technology of China, CHINA

## Abstract

Decidualization is a crucial process for successful embryo implantation and pregnancy in humans. Defects in decidualization during early pregnancy are associated with several pregnancy complications, such as pre-eclampsia, intrauterine growth restriction and recurrent pregnancy loss. However, the mechanism underlying decidualization remains poorly understood. In the present study, we performed a systematic analysis of decidualization-related genes using text mining. We identified 286 genes for humans and 287 genes for mice respectively, with an overlap of 111 genes shared by both species. Through enrichment test, we demonstrated that although divergence was observed, the majority of enriched gene ontology terms and pathways were shared by both species, suggesting that functional categories were more conserved than individual genes. We further constructed a decidualization-related protein-protein interaction network consisted of 344 nodes connected via 1,541 edges. We prioritized genes in this network and identified 12 genes that may be key regulators of decidualization. These findings would provide some clues for further research on the mechanism underlying decidualization.

## Introduction

In mammals, pregnancy begins with embryo implantation into uterus [1]. In some species such as humans and mice, invasive embryo implantation is accompanied by a rapid remodeling process in the stromal compartment of uterus known as decidualization. Upon decidualization, stromal cells undergo proliferation and subsequent differentiation into large epithelioid cells characterized by cytoplasmic accumulation of glycogen and lipid droplets, as well as an expansion of Golgi complex and rough endoplasmic reticulum [2, 3]. This process is marked by the secretion of decidual prolactin (PRL) and insulin-like growth factor binding protein 1 (IGFBP1) [4]. From a functional perspective, decidualization contributes to uterine angiogenesis and hemostasis during trophoblast invasion and placenta formation [5]. It also enables establishing maternal immunological tolerance to embryonic antigens [6]. Defects in decidualization during early pregnancy are associated with several pregnancy complications, such as pre-eclampsia, intrauterine growth restriction and recurrent pregnancy loss [7]. Therefore, it is imperative to gain a clear understanding of the molecular mechanism underlying decidualization in order to improve reproductive health.

In humans, decidualization is initiated spontaneously in the secretory phase of menstrual cycle [8]. If pregnancy is obtained, decidualization continues as the embryo undergoes implantation; otherwise, menstruation occurs. Most knowledge about human decidualization has come from studies using *in vitro* model systems. It is well established that decidualization can be induced in cultured endometrial stromal cells by incubation with progesterone after proper estrogen priming [9]. Decidualization is mediated by a gradual increase in intracellular cAMP level and addition of cAMP analogues leads to a boost of this process [10, 11]. The main advantage of the *in vitro* model systems is the ability to provide key information on a single cell type reaction. However, a cell growing as a layer in a dish does not have the complexity that a cell growing *in vivo* has. Most importantly, the uterus is a complex organ comprised of many cell types. Cultured stromal cells lack whole organ physiology and interacting microenvironment.

Because of ethical restrictions and experimental difficulties, it is not practical for *in vivo* study of decidualization in humans. Direct analysis of decidualization heavily relies on mice. Unlike humans, the decidual reaction in mice is an embryo-dependent process [8]. Decidualization begins shortly after the blastocyst attaches to the uterine luminal epithelium. Interestingly, hormonally primed uterus can be stimulated by mechanical means (e.g. sesame oil) to trigger decidualization in the absence of an embryo [12]. The mechanically decidualized endometrium, known as the deciduoma, is morphologically similar to the embryo-induced decidua, making it a good model of *in vivo* decidualization free of embryo contamination [13, 14]. A previous study has compared the global gene expression profiles between deciduoma and decidua [15]. Approximately 1,500 genes were differentially expressed by at least 1.2 folds. However, only 53 genes exhibited 2.5 folds or more, indicating that deciduoma is also similar to decidua at the transcriptome level.

Nevertheless, a comprehensive analysis of the molecular mechanism underlying decidualization is lacking. A wealth of information remains hidden within published research articles, the number of which is growing fast. Recently, the text mining methodology has been implemented, providing a necessary means to retrieve these data in an automated way [16]. Here we reported a systematic analysis of decidualization-related genes in humans and mice using text mining. Our study provides in-depth insights into the molecular mechanism underlying decidualization from a comparative aspect.

## Methods

### Text mining

The PubMed database was used. We conducted a search with the following combinations of query key words: “decidualization OR decidual OR decidua OR deciduas OR deciduoma OR decidualized OR decidualizing”. The search tag “[Title/Abstract]” was added after each key word. The relevant articles were retrieved in XML format. This format makes information extraction more precise owning to the use of enclosed contents within tag pairs. For each article, titles and abstract texts were fetched using the dom4j XML parser class in JAVA. Abstract texts were further divided into sentences through a sentence tokenizer implemented in LingPipe (Alias-I, Inc). Text mining was performed at the sentence level. Species names were parsed based on a lexicon [17]. All articles were classified into two categories according to species names mentioned in the texts: those studying human decidualization (including the monkey) and those studying mouse decidualization (including the rat). When no species name or multiple species names were detected, articles were classified manually.

**Fig 1:**
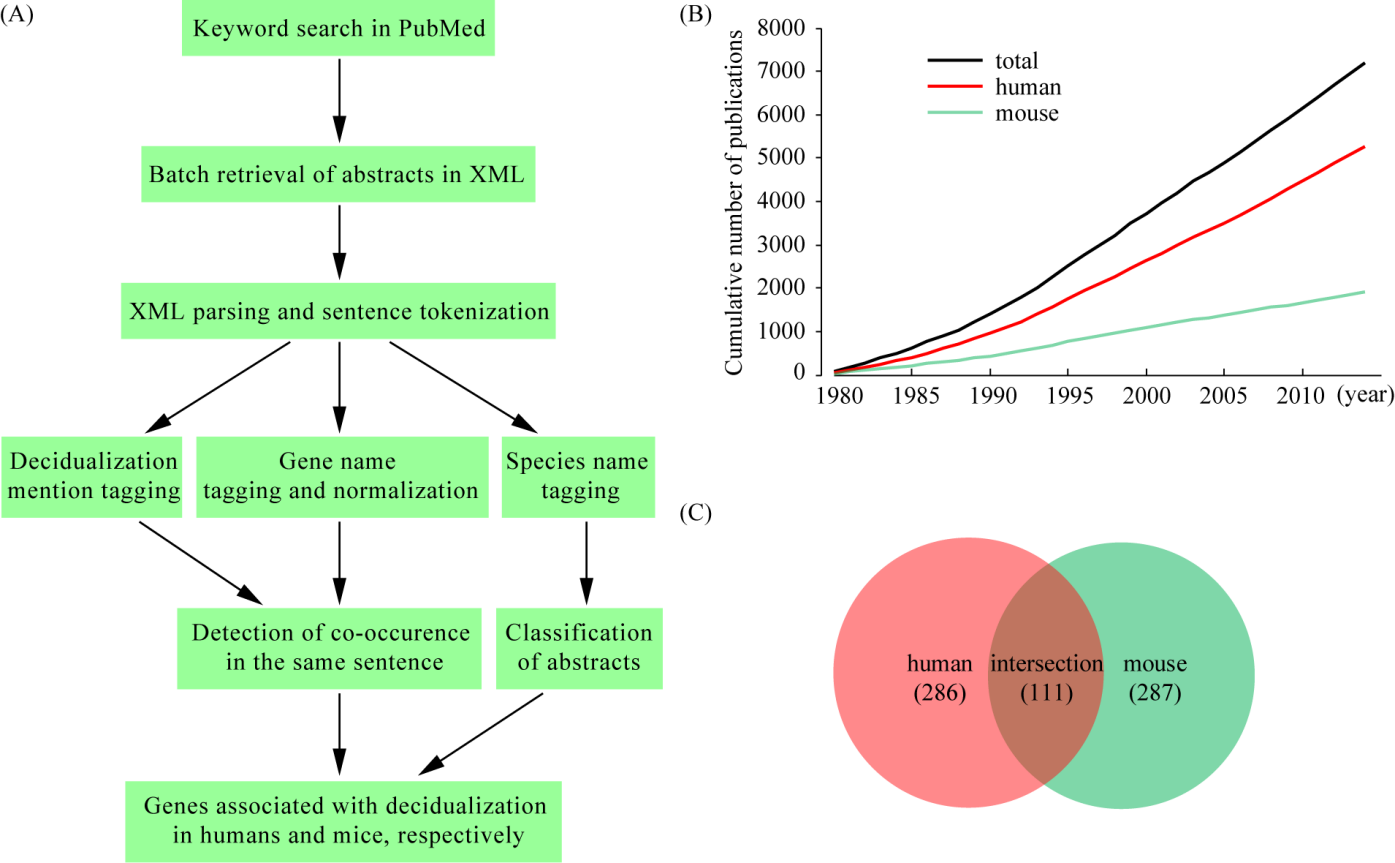
Systematic identification of genes associated with decidualization in humans and mice through text mining. (A) Overview of the text mining process. (B) The cumulative number of publications on decidualization. The PubMed database was used to identify publications related to decidualization from 1980-Jan to 2014-Aug. (C) Venn diagram comparing the gene sets associated with decidualization in humans and mice, respectively.

Gene mention recognition was performed using two different gene mention taggers, the hidden Markov model (HMM) tagger implemented in LingPipe and the ABNER tagger [18] based on a machine learning system of conditional random fields (CRF). Gene mentions detected by both taggers were merged. Because researchers name the genes in a highly variable manner, we built a gene synonym dictionary from Entrez gene database [19]. This dictionary was used for the gene name normalization process during which gene mentions were mapped to unique Entrez genes using exact string match. If multiple Entrez genes were linked to the same gene mention, the ambiguity was resolved manually. In order to reduce false positives, we required co-occurrence of decidualization mention and gene mention within a single sentence. In general, the abstract is sufficient for our text mining task, as it contains the most important findings of an article. However, articles on high throughput experiments often reveal a large number of genes which cannot be fully listed in the abstracts. For these articles, we downloaded full texts (as well as supplementary files if needed) and extracted gene mentions by hands. Finally, we compiled two gene sets: one is associated with human decidualization and the other one is associated with mouse decidualization. To ensure accurate and complete recording, each gene was checked manually and additional references were provided if possible. A flow chart illustrating the text mining procedure is shown in **Fig 1A**.

### Gene ontology (GO) analysis

GO enrichment analysis was performed by using BiNGO 2.3 with the GOslim dataset [20]. To test for enrichment, a hypergeometric test was conducted followed by Benjamini and Hochberg multiple test correction. The adjusted p-value < 0.05 was used as the significance threshold to identify enriched categories.

### Pathway analysis

To rank the importance of pathways involved in decidualization, we calculated enrichment p-values followed by Benjamini and Hochberg multiple test correction through DAVID online tools 6.7 [21]. The significance threshold was set at 0.05. To visualize the JAK-STAT pathway, decidualization-related genes mapped to this pathway were retrieved. Gene dependencies were determined using the R package KEGGSOAP [22]. The pathway was rendered in Cytoscape [23].

### Construction of protein-protein interaction (PPI) network

The decidualization-related genes were cross-referenced with PINA2 database [24]. The PINA2 database provides integrated and up-to-date protein-protein interactions available in IntAct [25], BioGRID [26], MINT [27], DIP [28], HPRD [29] and MIPS [29]. To query the PINA2 database, species was restricted to human and mouse. Interactions determined by both low throughput and high throughput experiments were included. The PPI network was illustrated in Cytoscape with the nodes representing genes and edges representing their interactions. Topological parameters were analyzed by NetworkAnalyzer [30]. The edges in the network were treated as undirected. The degree of a node was the number of its directly connecting neighbors in the network. Genes were prioritized by the decidualization impact factor (DIF), which is defined as degree times the number of publications for each gene. The threshold of DIF was the mean plus two standard deviations.

## Results

### Identification of genes associated with decidualization using text mining

We run a key word search in the PubMed database for articles related to decidualization and obtained 7,185 entries as a result (from 1980-Jan to 2014-Aug). Abstracts of these articles were downloaded and processed through a text mining pipeline shown in **Fig 1A**. All articles were classified into two categories according to species names mentioned in the texts: those studying human decidualization and those studying mouse decidualization. Cumulative distribution analysis indicated that the number of articles published on decidualization is growing linearly in recent years: on average, 151 articles per year on human decidualization and 55 articles per year on mouse decidualization (**Fig 1B**). From these articles, we extracted genes via text mining. In the end, we compiled a complete list of decidualization-related genes, 286 genes for human decidualization and 287 genes for mouse decidualization (**S1 Table**). Cross-species comparison revealed that 111 genes were shared by both humans and mice (**Fig 1C**).

### Gene ontology (GO) analysis

**Fig 2:**
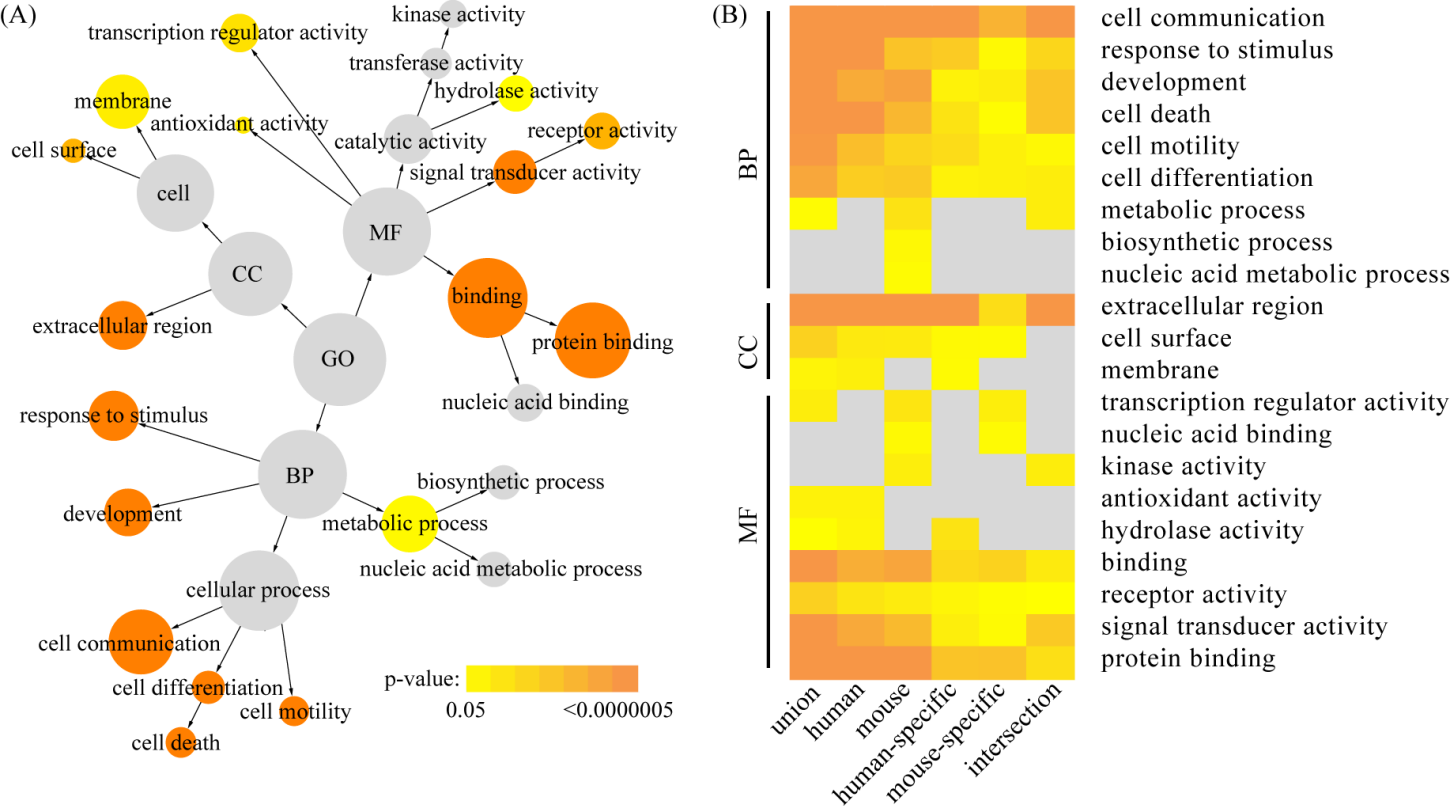
Gene ontology (GO) enrichment analysis of decidualization-related genes. (A) The union of human and mouse gene sets were analyzed using BiNGO software. Significantly enriched GOslim categories were highlighted with different colors representing different levels of significance. The size of each circle is correlated to the number of genes. (B) Comparative GO enrichment analysis for species-specific gene sets arranged in the biological process category (BP), the cellular component category (CC) and the molecular function category (MF), respectively. The analysis was applied to human and mouse gene sets, as well as 4 additional gene sets generated by set operations (union, intersection and difference) between them. The columns represent different gene sets, while the rows represent statistically significant GO terms.

All 462 decidualization-related genes (286 genes for humans and 287 genes for mice) were functionally categorized based on gene ontology (GO) annotation terms using BiNGO software. Enrichment analysis revealed that a total of 17 GO terms exhibited significance as overrepresented terms (p < 0.05). In the biological process category, 7 GO terms, namely cell communication, response to stimulus, development, cell death, cell motility, cell differentiation and metabolic process, were found to be significantly enriched. GO terms related to extracellular region, cell surface and membrane were significantly enriched under the cellular component category. Enriched GO terms in the molecular function category were transcription regulator activity, antioxidant activity, hydrolase activity, binding, receptor activity, signal transducer activity and protein binding. The hierarchical organization of these GO terms is shown in **Fig 2A**, together with the significance of enrichment indicated by different colors.

To assess the overall functional similarities and differences of decidualization between humans and mice, we tested the species-specific gene sets for enrichment of GO terms (**Fig 2B**). We found that the majority of GO terms enriched in species-specific gene sets were common to each other. However, several GO terms were uniquely enriched. The most notably enriched GO terms unique to mice were nucleic acid metabolic process and biosynthetic process in the biological process category and transcription regulator activity and nucleic acid binding in the molecular function category. As for humans, the most notably unique GO terms were hydrolase activity and antioxidant activity in the molecular function category.

### Pathway analysis

**Fig 3:**
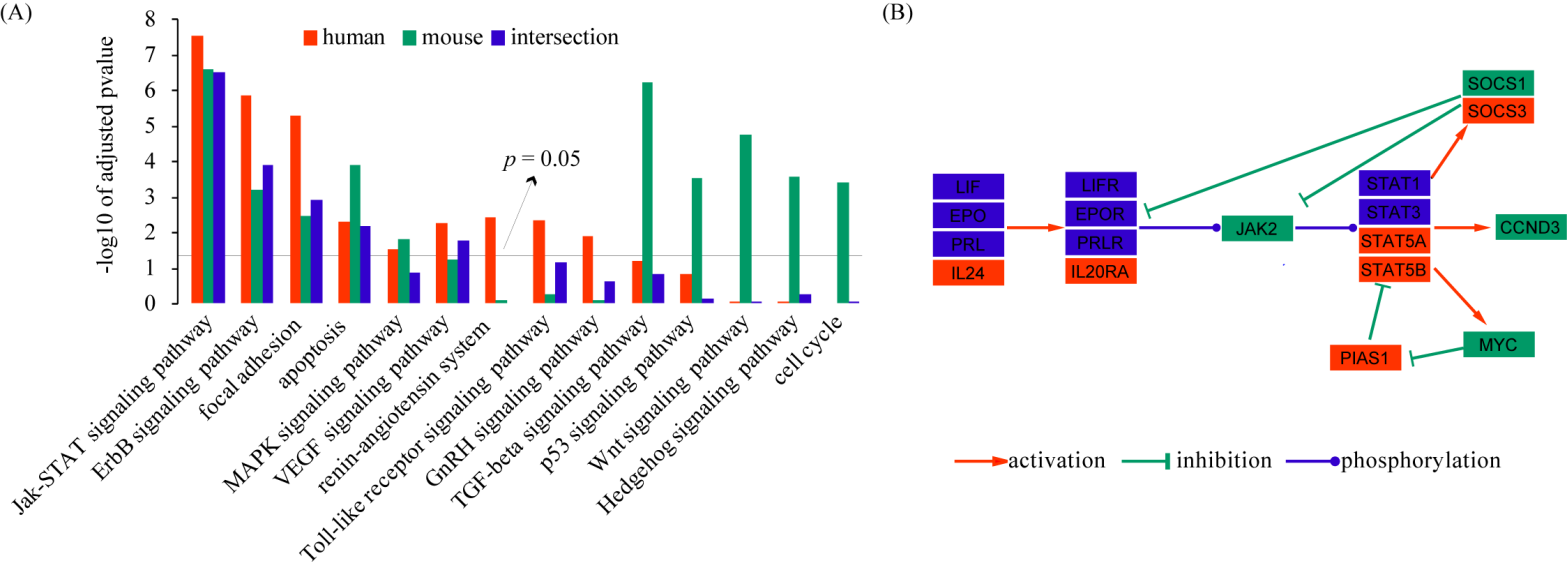
Pathway enrichment analysis of decidualization-related genes. (A) The figure shows the significantly enriched pathways identified by using DAVID online tools. The bars represent the enrichment p-value at logarithmic scale. (B) Visualization of the LIF-STAT signaling pathway. Nodes represent genes. The node color indicates the status of the gene as specific to human (red), specific to mouse (green), or shared by both (blue). Edges represent gene dependences derived from KEGG pathway database. Genes without a direct interaction with others are not included. This graph is generated using the Cytoscape software.

In addition to GO analysis, we also performed pathway analysis by using DAVID online tools. Unlike GO, which only contains gene lists for different functional categories, pathway database also stores the information of gene dependencies in each pathway. In the present study, all decidualization-related genes were mapped to KEGG pathways. A total of 5 pathways, namely Jak-STAT signaling pathway, ErbB signaling pathway, focal adhesion, apoptosis and MAPK signaling pathway, were significantly enriched in both human and mouse gene sets (p < 0.05) (**Fig 3A**). The enrichment of VEGF signaling pathway, renin-angiotensin system, Toll-like receptor signaling pathway and GnRH signaling pathway was unique to the human gene set, whereas TGF-beta signaling pathway, p53 signaling pathway, Wnt signaling pathway, Hedgehog signaling pathway and cell cycle was enriched only in the mouse gene set (**Fig 3A**). Based on enrichment p-values, the most highly overrepresented pathway went to the LIF-STAT pathway. In this pathway, we identified 18 genes, of which 6 are specific to humans, **4** are specific to mice, and 8 are shared by both (**Fig 3B**). The LIF-STAT pathway is known to play an important role during decidualization in both humans [31] and mice [32–34].

### Gene prioritization by protein-protein interaction (PPI) network analysis

**Fig 4:**
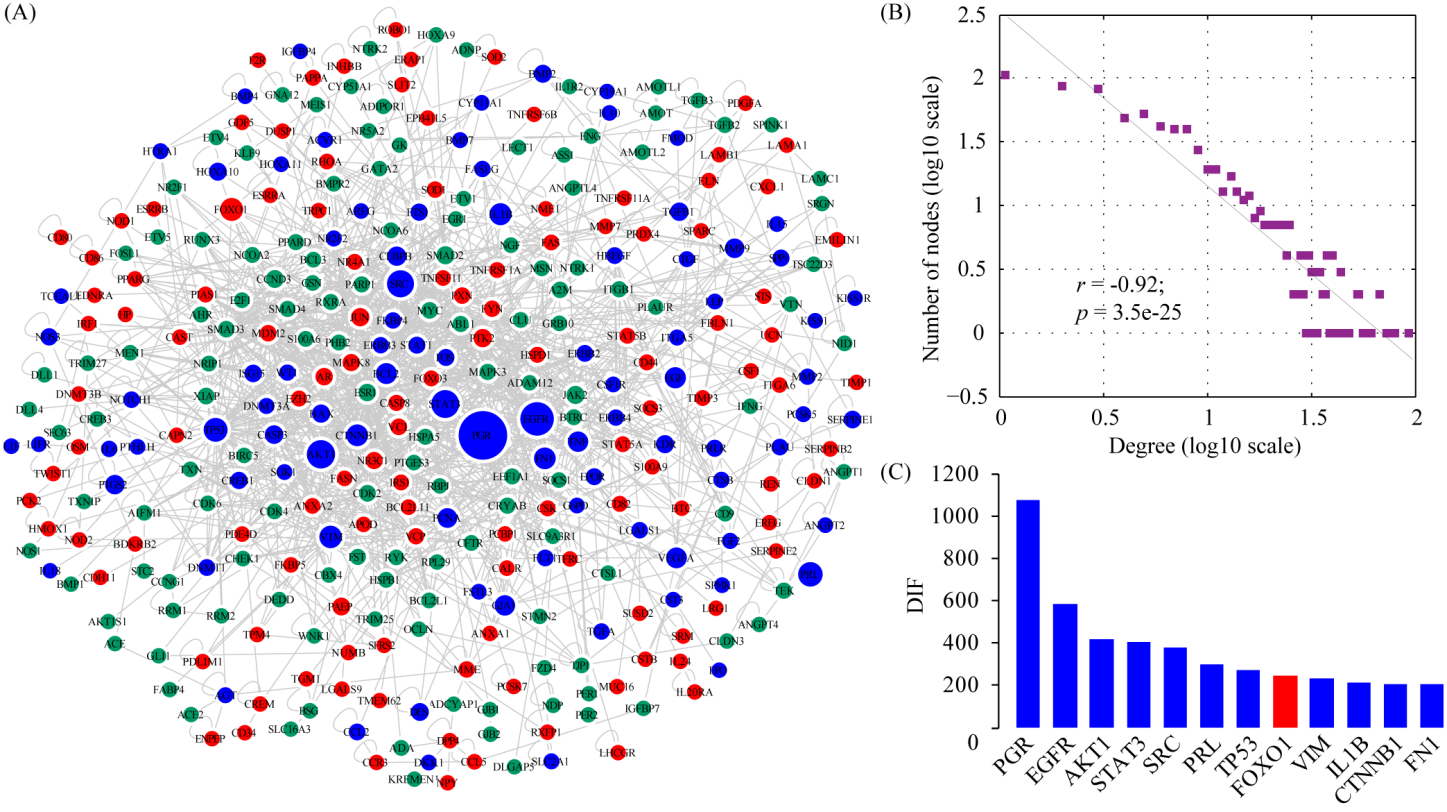
Gene prioritization by protein-protein interaction (PPI) network analysis. (A) The structure of the PPI network of decidualization-related genes. Nodes are color-coded (red, human-specific; green, mouse-specific; blue, shared by both) and the diameter of each node is proportional to its decidualization impact factor (DIF) value. (B) Degree distribution of the PPI network. The degree distribution follows a power law distribution. (C) Bar plot showing the DIF values for all selected genes with DIF values exceeding the mean plus two standard deviations.

A genome-wide protein-protein interaction (PPI) network was constructed by merging up-to-date protein-protein interactions available in IntAct [25], BioGRID [26], MINT [27], DIP [28], HPRD [35] and MIPS [29]. The network related to decidualization was generated by mapping decidualization-related genes to the genome-wide PPI network. The decidualization network consisted of 344 nodes connected via 1,541 edges (**Fig 4A**). Topological analysis showed that the network follows a power-law distribution (**Fig 4B**) and therefore is a scale-free small world network [36]. Networks of this type have the particular feature that some nodes are highly connected compared with others. The highly connected nodes, also known as hub genes, represent functionally important genes in the network. Taking the number of publications into consideration, we prioritized genes by the decidualization impact factor (DIF), which is defined as degree times the number of publications for each gene. Using a defined threshold value of 193, we identified 12 genes (**Fig 4C**). Interestingly, all these genes (PGR, EGFR, AKT1, STAT3, SRC, PRL, TP53, VIM, IL1B, CTNNB1 and FN1), except FOXO1 which was specific to human, were shared by both humans and mice, suggesting that the core gene network underlying decidualization is conserved between species.

## Discussion

In the present study, we attempted to compile a complete list of genes involved in decidualization. In recent years, high-throughput transcriptomic and proteomic approaches make it possible for studying the expression levels of thousands of genes and proteins simultaneously. Global gene or protein expression changes upon decidulization have been determined by independent groups [37–41]. However, little consistency is observed in these studies. In general, due to high technical variability and high dimensional size, the results of high-throughput data are not highly reliable. Moreover, the different choices of platforms and statistical criteria make it even more difficult to compare between studies [42]. On the other hand, a wealth of information remains hidden within published research articles using conventional gene-by-gene methods. Recently, the text mining methodology has been implemented, providing a necessary means to retrieve these data in an automated way [16]. Here we performed a text mining analysis of decidualization-related genes. We identified 286 genes for human decidualization and 287 genes for mouse decidualization, respectively. Considering the large body of literature we analyzed, our result may have reasonably good coverage of all decidualization-related genes.

Regardless of species, we found that 462 genes were associated with decidualization. Of all these genes, only 24 genes are down-regulated during decidualization, indicating that existing studies are mainly focused on up-regulated genes. Interestingly, PGR and HOXA10 are among the down-regulated genes, although they are absolutely needed for decidualization. The most reliable evidence that a gene is involved in decidualization relies on gene knockout in mice. However, so far there are only 39 genes reported to cause impaired decidualization in gene knockout experiments (shown in **S1 Table** with references). Due to the small population, these genes may be highly biased and unsuitable to study the general mechanism of decidualization. Alternatively, in this study we identified genes that are expressed or functional in the decidual tissues. Based on GO analysis, a total of 17 terms were significantly enriched, including biological processes involved in cell communication, response to stimulus, development, cell death, cell motility, cell differentiation and metabolic process, cellular components related to extracellular region, cell surface and membrane, and molecular functions associated with transcription regulator activity, antioxidant activity, hydrolase activity, binding, receptor activity, signal transducer activity and protein binding. Additionally, our study also revealed 14 enriched pathways. Of particular interest was the LIF-STAT pathway, in which 18 genes were identified (6 are specific to humans, 4 are specific to mice, and 8 are shared by both). LIF null female mice are infertile due to implantation failure [43]. Knockout of STAT3 in mice leads to embryonic lethality [44]. Conditional ablation of STAT3 in mouse uterus impairs uterine receptivity and decidualization [32–34]. In cultured human endometrial stromal cells, LIF and STAT3 have been shown to be important regulators of decidualization [31]. Taken together, we conclude that LIF-STAT pathway plays a consensus role during decidualization in both humans and mice, although species-specific fine-tuning of certain components may exist.

The mouse model serves as an important experimental system for biomedical science. To date, various studies have found similarities between humans and mice at the molecular level [45–47]. In the present study, we examined the similarities and differences in decidualization between these two species. At the gene level, among the 286 genes that were associated with human decidualization, 111 genes or 38.8% were also discovered to be associated with the same process in mice. Based on enrichment test, the majority of enriched GO terms were shared between the two species, suggesting that functional categories are more conserved than individual genes. We speculate that a similar set of functional categories may be required by decidualization in humans and mice, but each functional category can be implemented by alternative genes in these two species. This may explain why only a small portion of consensus is needed at the gene level. Nevertheless, we did observe that several GO terms were uniquely enriched in humans or mice. The most notably enriched GO terms unique to mice were nucleic acid metabolic process and biosynthetic process in the biological process category and transcription regulator activity and nucleic acid binding in the molecular function category. As for humans, the most notably unique GO terms were hydrolase activity and antioxidant activity in the molecular function category. These data suggest that decidualization in humans and mice is not congruent in some aspects and such differences should be considered in the context of clinical translation.

Enrichment analysis treats GO and pathway terms as segregated entities, ignoring the effect of shared genes. In addition, only a portion of genes in genome are assigned to GO or pathway terms due to incomplete curation. Alternatively, protein-protein interaction (PPI) network, which offers a better coverage of the whole genome, is able to provide important clues about gene functions. In the present study, we constructed a decidualization-related gene network by using protein-protein interaction data available in IntAct [25], BioGRID [26], MINT [27], DIP [28], HPRD [35] and MIPS [29]. This network consisted of 344 nodes connected via 1,541 edges. We prioritized genes in this network by the decidualization impact factor (DIF), which is defined as degree times the number of publications for each gene. Using a defined threshold value of 193, we identified 12 genes. Interestingly, all these genes (PGR, EGFR, AKT1, STAT3, SRC, PRL, TP53, VIM, IL1B, CTNNB1 and FN1), except FOXO1 which was specific to human, were shared by both humans and mice, suggesting that the core gene network underlying decidualization is conserved between species. As expected, progesterone receptor gene (PGR) turned out the most important gene in the network. Undoubtedly, decidualization is a progesterone-dependent process and progesterone exerts its effects via its nuclear receptor PGR [48, 49]. Besides PGR, we also indentified another 3 transcription factors, TP53, STAT3 and FOXO1. It has been well established that mouse and human fibroblasts can be reprogrammed to undifferentiated pluripotent stem cells with a combination of 4 transcription factors (Oct4, Sox2, Klf4 and Myc) [50]. Recent studies have shown that transcription factors could also reprogram somatic cell into another type of defined somatic cells without the undifferentiated state. For example, a combination of only 3 transcription factors, Ascl1, Brn2 and Myt1l, can efficiently convert mouse embryonic and postnatal fibroblasts into functional neurons [51]. A combination of 3 transcription factors (Ngn3, Pdx1 and Mafa) is able to reprogram pancreatic exocrine cells into insulin-secreting β-cells [52]. Fibroblasts can be directly reprogrammed into functional cardiomyocytes by 3 defined transcription factors, Gata4, Mef2c, and Tbx5 [53]. It has also been demonstrated that enforced expression of a single transcription factor (Foxn1) is sufficient to reprogram fibroblasts into functional thymic epithelial cells [54]. Based on these well-documented examples, we hypothesize that combinatorial expression of decidua-specific transcription factors could directly convert fibroblasts into decidual cells in the absence of hormones. Once these induced decidual cells (iDCs) are generated, they might help in understanding the mechanism of decidualization and open the possibility of therapeutic use. In the present study, we prioritized a total of 4 transcription factors using PPI network analysis. In theory, these transcription factors are drivers of molecular and morphological changes during decidualization, therefore providing an ideal starting pool of candidate transcription factors for generating iDCs.

In summary, we have reported here the first systematic analysis of the molecular mechanism underlying decidualization in humans and mice using a text mining approach. We examined the similarities and differences between these two species at the gene, gene ontology, pathway and network levels. Our study provides a valuable resource for in-depth understanding of the molecular mechanism underlying decidulization.

## Supporting Information

S1 Table A complete list of decidualization-related genes. (XLS)
